# Secondary analysis of the WOMAN trial to explore the risk of sepsis after invasive treatments for postpartum hemorrhage

**DOI:** 10.1002/ijgo.12860

**Published:** 2019-06-05

**Authors:** Laura Cornelissen, Susannah Woodd, Haleema Shakur‐Still, Bukola Fawole, Shehla Noor, Saturday Etuk, Adesina Lawrence Akintan, Rizwana Chaudhri, Ian Roberts

**Affiliations:** ^1^ London School of Hygiene and Tropical Medicine London UK; ^2^ Department of Obstetrics and Gynecology UZ Leuven Leuven Belgium; ^3^ Department of Infectious Disease Epidemiology London School of Hygiene and Tropical Medicine London UK; ^4^ Clinical Trials Unit London School of Hygiene and Tropical Medicine London UK; ^5^ Department of Obstetrics and Gynecology National Institute of Maternal and Child Health College of Medicine University of Ibadan Ibadan Nigeria; ^6^ Department of Obstetrics and Gynecology Ayub Medical and Teaching Institute Abbottabad Pakistan; ^7^ Department of Obstetrics and Gynecology College of Medical Sciences University of Calabar Calabar Nigeria; ^8^ Department of Obstetrics and Gynecology, Mother and Child Hospital Akure Nigeria; ^9^ Department Obstetrics and Gynecology Rawalpindi Medical University Rawalpindi Pakistan

**Keywords:** Antibiotic prophylaxis, Brace sutures, Intrauterine tamponade, Maternal morbidity, Maternal mortality, Maternal sepsis, Peripartum infections, Postpartum hemorrhage

## Abstract

**Objective:**

To examine the association between the use of invasive treatments for postpartum hemorrhage and the risk of sepsis and severe sepsis.

**Methods:**

Secondary data analysis of the WOMAN randomized controlled trial, including 20 060 women with postpartum hemorrhage in 21 countries. Logistic regression with random effects was used.

**Results:**

The cumulative incidence was 1.8% for sepsis and 0.5% for severe sepsis. All‐cause mortality was 40.4% in women with severe sepsis versus 2.2% for women without. After adjusting for bleeding severity and other confounders, intrauterine tamponade, hysterectomy, and laparotomy increased the risk of sepsis (aOR 1.77 [95% CI 1.21–2.59], *P*=0.004; aOR 1.97 [95% CI 1.49–2.65], *P*<0.001; and aOR 6.63 [95% CI 4.29–10.24], *P*<0.001, respectively) and severe sepsis (aOR 2.60 [95% CI 1.47–4.59], *P*=0.002; aOR 1.97 [95% CI 0.83–2.46], *P*=0.033; and aOR 5.35 [95% CI 2.61–10.98], *P*<0.001, respectively).

**Conclusion:**

In this secondary data analysis, certain invasive treatments for postpartum hemorrhage appear to increase the risk of sepsis. Further research is needed to confirm this finding and investigate the role of prophylactic antibiotics during these procedures. The harms and benefits of such interventions must be carefully weighed, both in treatment guidelines and during individual patient management.

**Trial Registration: ISRCTN76912190:**

## Abstract 2

Invasive treatments for postpartum hemorrhage, particularly laparotomy and intrauterine tamponade, increase the risk of sepsis.

## 1 INTRODUCTION

Every day, approximately 830 women die worldwide as a result of pregnancy and childbirth.^1^ Most maternal deaths (99%) occur in low‐ and middle‐income countries (LMICs),^1^ and are caused by hemorrhage (27%), hypertensive disorders (14%), or sepsis (10.7%).^2^ Classifying death by a single primary cause, however, misses the potentially important contribution of other morbidities and their interactions.

Imprecise and varying definitions of maternal sepsis have been used for many years. In 2017, WHO responded by proposing a definition of maternal sepsis as a “life‐threatening condition defined as organ dysfunction resulting from infection during pregnancy, childbirth, post‐abortion, or postpartum period”.^3^ This change is in line with the new Sepsis‐3 definition for the general population.^4^ Specific criteria for identification are yet to be developed.^3^

Risk factors for peripartum infections include pre‐existing maternal conditions (obesity, diabetes, malnutrition, severe anemia) and factors related to childbirth (cesarean delivery, prolonged rupture of membranes, multiple vaginal examinations, placental retention).^5^, ^6^, ^7^ Interest in postpartum hemorrhage (PPH) as a risk factor for sepsis has recently been sparked by an association found in population‐based studies in high‐income countries.^8^, ^9^, ^10^, ^11^ It is possible that invasive treatments for PPH, such as intrauterine tamponade and hysterectomy, could increase the risk of infection by introducing (vaginal) bacteria into the uterus and abdomen.

Despite the possible infectious risk, antibiotic prophylaxis is rarely and inconsistently mentioned in PPH treatment guidelines; the American College of Obstetricians and Gynecologists 12 and the Royal College of Obstetricians and Gynaecologists in the UK 13 make no mention at all. WHO guidelines only recommend prophylactic antibiotics for manual removal of the placenta.^14^ This difference in guidelines indicates a lack of evidence in the area.

The World Maternal Antifibrinolytic (WOMAN) trial recruited women with PPH in 21 countries.^15^ Tranexamic acid was shown to reduce mortality from hemorrhage by 19%. Sepsis was noted to be an important complication. The aim of the present study was to examine this large dataset to determine the association between invasive treatment for PPH and sepsis.

## 2 MATERIALS AND METHODS

We conducted a secondary analysis of all women recruited to the WOMAN trial to assess the association between invasive treatment for PPH and sepsis. Our primary outcome was sepsis, defined by the study authors at the onset of the trial. Our secondary outcome was sepsis with organ dysfunction. Main exposures were manual removal of placenta, hysterectomy, brace sutures, artery ligation (individually or a combination of the uterine artery, ovarian artery, internal iliac artery), intrauterine tamponade, and “laparotomy for other reasons.”

The WOMAN trial is a randomized, double‐blind, placebo‐controlled trial to evaluate the effect of tranexamic acid on mortality or hysterectomy in women with PPH. Details have been described elsewhere.^15^ In brief, 20 060 women were recruited in 193 facilities in 21 countries. Women over 16 years were eligible if they had a clinical diagnosis of PPH (estimated blood loss >500 mL after vaginal delivery or >1000 mL after cesarean, or any blood loss sufficient to compromise hemodynamic stability). Baseline characteristics included maternal age, place and type of delivery, complete expulsion of the placenta, primary cause of bleeding, and use of uterotonics. Hemodynamic instability at entry was based on clinical signs (e.g. low blood pressure, tachycardia, falling urine output). The primary outcome was a composite of death from all causes or hysterectomy. Secondary outcomes included complications (renal failure, cardiac failure, respiratory failure, hepatic failure, sepsis, and seizures) and surgical interventions to treat hemorrhage (intrauterine tamponade, embolization, brace sutures, arterial ligation, hysterectomy, and laparotomies done for other reasons). Outcomes were recorded from medical records at time of death, at discharge, or 42 days postpartum, whichever occurred first.

Sepsis in the WOMAN trial was defined as infection plus systemic inflammatory response, in line with the previous adult definition of sepsis that was still in use when inclusion started in 2010.^16^ In the present analysis, a new variable “severe sepsis” was created as sepsis plus organ dysfunction. Note that this “severe sepsis” variable equals the term sepsis as defined by the 2016 Sepsis‐3 consort.^4^ Organ dysfunction in the WOMAN trial was diagnosed as follows: renal failure required either a rise in serum creatinine of greater than or equal to 26 μmol/L (0.29 mg/dL) within 48 hours, rise in serum creatinine of 50% or greater known or presumed to have occurred within the past 7 days, urine output less than 0.5 mL/kg/h for more than six consecutive hours, or (in those with pre‐existing renal disease) a serum creatinine rise of 200% or more from index serum creatinine or serum creatinine increased to 350 μmol/L (4 mg/dL). Cardiac failure required the presence of typical signs or symptoms (e.g. orthopnea, hepatojugular reflux) or a reduced left ventricular ejection fraction, relevant structural heart disease, or diastolic dysfunction. Respiratory failure required a partial pressure of oxygen less than 60 mm Hg (8.0 kPa) on room air, sea level. Hepatic failure required deterioration in liver function with changes in mental status and coagulopathy. No other types of organ dysfunction were recorded (Table S1 holds “guidance on diagnosing complications”).

Percentages and medians were used to describe the data. Risk factors for sepsis were examined using logistic regression with random effects to account for clustering by facility. Main exposures were manual removal of placenta, hysterectomy, brace sutures, artery ligation (individually or a combination of the uterine artery, ovarian artery, internal iliac artery), intrauterine tamponade, and “laparotomy for other reasons.” We built a comprehensive model including all possible confounders (age, type of delivery, hospital delivery, primary cause of hemorrhage, and markers of bleeding severity: estimated blood loss, systolic blood pressure, and hemodynamic instability). New organ dysfunctions are thought to result from sepsis rather than preceding it and were therefore excluded as risk factors. The same model was rerun with severe sepsis as the outcome.

Comprehensive logistic regression models with random effects were also used to assess the effect of (severe) sepsis on all‐cause mortality. A priori confounders were age, type of delivery, signs of hemodynamic instability, place of delivery, prophylactic use of uterotonics, and primary cause of hemorrhage. Organ dysfunction lies on the causal pathway and was excluded.

All analyses were carried out using Stata version 14 (StataCorp, College Station, TX, USA). Odds ratios, 95% confidence intervals, and *P* values from likelihood ratio tests are presented. We considered a *P* value of <0.05 to be significant.

The initial study was registered under ISRCTN76912190. Approval was obtained from local ethics committees and the ethics committee of the London School of Hygiene and Tropical Medicine (LSTHM). Ethical approval for secondary data analysis was granted by the ethics committee of LSTHM under ref number 13400. The WOMAN trial obtained consent from its participants in line with the procedure described in the protocol. No additional patient information was collected for the present study.

## 3 RESULTS

A total of 20 060 women with PPH were included in the WOMAN trial, with a mean age of 28 years. Data on sepsis were available for 20 018 (42 missing, 0.2%). During the period of observation, 483 women died (all‐cause mortality 2.4%). There were 365 cases of sepsis reported (1.8%), of which 104 met our criteria for severe sepsis (0.5% of the total population). Most women delivered in hospital (87.9%) and almost all received prophylactic uterotonics (96.2%). Almost one‐third (29.1%) of women were delivered by cesarean. Data collection was nearly complete for most variables. Table 1 presents an overview of the population characteristics and missing values.

**Table 1:** Characteristics of the study population and univariate analysis of risk factors for sepsis and severe sepsis (n=20 018)

Characteristics	No. of women	Cases of sepsis (%)	Crude OR (95% CI)^a^	Cases of severe sepsis (%)	Crude OR (95% CI)^a^
Type of delivery					
Vaginal	14 189	193 (1.36)	1	63 (0.44)	1
Cesarean	5824	170 (2.91)	2.51 (2.00–3.16)	41 (0.70)	1.80 (1.17–2.77)
Missing	5	2		0	
Age group, y					
≤20	1978	31 (1.56)	1.10 (0.71–1.70)	8 (0.40)	1.47 (0.62–3.50)
20–25	4864	78 (1.60)	1	17 (0.35)	1
>25–30	6794	109 (1.60)	1.03 (0.76–1.39)	33 (0.49)	1.60 (0.88–2.94)
>30–40	6066	140 (2.30)	1.38 (1.03–1.85)	43 (0.71)	2.34 (1.30–4.22)
>40	309	7 (2.25)	1.52 (0.67–3.44)	3 (0.97)	3.32 (0.91–12.13
Missing	7	0		0	
Hospital delivery					
Yes	17 587	294 (1.67)	1	82 (0.47)	1
No	2428	70 (2.88)	1.43 (1.07–1.92)	22 (0.91)	1.69 (1.02–2.86)
Unknown	3	1		0	
Primary cause of hemorrhage					
Atony	12 759	165 (1.29)	1	50 (0.39)	1
Trauma	3681	88 (2.38)	1.60 (1.21–2.10)	22 (0.60)	1.17 (0.69–1.98)
Placenta previa/accreta	1874	57 (3.04)	1.60 (1.15–2.22)	11 (0.59)	0.78 (0.39–1.56)
Other	1454	45 (3.09)	1.69 (1.19–2.41)	19 (1.31)	2.17 (1.23–3.88)
Unknown	250	10		2	
Prophylactic uterotonics					
Yes	19 265	341 (1.77)	1	94 (0.49)	1
No	269	14 (5.19)	2.25 (1.25–4.06)	6 (2.23)	3.84 (1.56–9.42)
Unknown	484	10		4	
Hemodynamic instability					
No	8194	66 (0.80)	1	12 (0.15)	1
Yes	11 823	299 (2.52)	3.17 (2.37–4.24)	92 (0.78)	6.39 (3.36–12.13)
Unknown	1	0		0	
Estimated blood loss, mL					
≤1000	10 402	93 (0.89)	1	28 (0.27)	1
1001–2000	8284	206 (2.48)	3.01 (2.29–3.95)	50 (0.60)	3.20 (1.87–5.48)
>2000	1330	66 (4.94)	5.75 (4.02–8.21)	26 (1.95)	10.21 (5.39–19.35)
Unknown	2	0		0	
Systolic blood pressure, mm Hg					
≥100	12 097	156 (1.29)	1	31 (0.26)	1
90–99	4081	65 (1.59)	1.24 (0.92–1.70)	21 (0.51)	1.93 (1.08–3.45)
<90	3835	144 (3.74)	3.07 (2.39–3.94)	52 (1.36)	6.03 (3.72–9.79)
Unknown	5	0		0	
Hysterectomy					
No	18 997	293 (1.54)	1	79 (0.42)	1
Yes	1020	72 (7.06)	4.55 (3.41–6.07)	25 (2.45)	5.87 (3.56–9.66)
Unknown	1	0		0	
Manual placenta removal					
No	18 138	302 (1.67)	1	89 (0.49)	1
Yes	1879	63 (3.35)	1.42 (1.05–1.93)	15 (0.80)	1.18 (0.66–2.12)
Unknown	1	1		0	
Intrauterine tamponade					
No	18 583	310 (1.67)	1	73 (0.39)	1
Yes	1434	55 (3.84)	1.97 (1.38–2.82)	31 (2.13)	2.955 (1.74–5.02)
Unknown	1	1		0	
Embolization					
No	19 994	363 (1.82)	1	102 (0.51)	1
Yes	23	2 (8.7)	6.39 (1.23–33.35)	2 (8.7)	40.70 (7.00–236.52)
Unknown	1	1		0	
Laparotomy					
No	19 808	323 (1.63)	1	88 (0.44)	1
Yes	209	42 (20.1)	13.38 (8.98–19.93)	16 (7.66)	16.22 (8.66–30.32)
Unknown	1	1		0	
Brace sutures					
No	19 467	339 (1.74)	1	91 (0.47)	1
Yes	550	26 (4.73)	2.67 (1.72–4.16)	13 (2.36)	4.35 (2.27–8.31)
Unknown	1	1		0	
Artery ligation					
No	19 538	328 (1.68)	1	83 (0.42)	1
Yes	479	37 (7.72)	3.91 (2.63–5.82)	21 (4.38)	6.76 (3.85–11.87)
Unknown	1	1		0	

a Logistic regression with random effects to account for clustering.

In univariate analysis, all surgical interventions to treat PPH showed evidence of an association with sepsis (Table 1). Sepsis occurred more than twice as often in women who underwent cesarean delivery (OR 2.51; 95% CI, 2.00–3.16; *P*<0.001). A higher proportion of hemodynamically unstable women developed sepsis.

After adjusting for bleeding severity and other confounding factors, brace sutures, manual placenta removal, and artery ligation were no longer associated with sepsis. Strong evidence (*P*<0.001) remained for associations between sepsis and hysterectomy (aOR 1.97; 95% CI, 1.49–2.65) and laparotomy (aOR 6.63; 95% CI, 4.29–10.24), but effect sizes were smaller than in univariate analysis. The estimated effect of intrauterine tamponade (aOR 1.77; 95% CI, 1.21–2.59]) remained essentially unchanged (*P*=0.004).

The model for severe sepsis adjusted for the same confounders as above, including bleeding severity, but contained fewer events (104 cases of severe sepsis). The confidence intervals are wider, but the main results remain similar. The risk factors associated with severe sepsis were hysterectomy (aOR 1.97; 0.83–2.46; *P*=0.033), intrauterine tamponade (aOR 2.60; 95% CI, 1.47–4.59; *P*=0.002), laparotomy (aOR 5.35; 95% CI, 2.61–10.98; *P*<0.001), and artery ligation (aOR 2.50; 1.28–4.89; *P*=0.010) (Table 2).

**Table 2:** Multivariate analysis of risk factors for sepsis, corrected for clustering using random effects (n=19 752)

Risk factors	Sepsis			Severe sepsis		
	aOR^a^	CI	*P* value^b^	aOR^a^	CI	*P* value^b^
Hysterectomy	1.97	1.49–2.65	<0.001	1.97	0.83–2.46	0.033
Manual placenta removal	1.30	0.92–1.83	0.139	0.90	0.46–1.74	0.750
Intrauterine tamponade	1.77	1.21–2.59	0.004	2.60	1.47–4.59	0.002
Laparotomy	6.63	4.29–10.24	<0.001	5.35	2.61–10.98	<0.001
Brace sutures	1.09	0.66–1.81	0.7369	1.36	0.62–2.99	0.454
Artery ligation	1.48	0.94–2.34	0.098	2.50	1.28–4.89	0.010
Age, y			0.891			0.652
20–25 y	1			1		
25–30 y	0.93	0.67–1.27		1.36	0.72–2.56	
30–40 y	1.02	0.75–1.39		1.63	0.87–3.07	
>40	0.76	0.30–1.91		1.37	0.32–5.79	
≤20	1.09	0.69–1.73		1.40	0.56–3.46	
Cesarean delivery	1.99	1.49–2.65	<0.001	1.43	0.83–2.46	0.197
Estimated blood loss, mL			0.006			0.139
≤1000	1			1		
1001–2000	1.65	1.21–2.26		1.66	0.89–3.10	
>2000	1.53	0.99–2.40		2.17	0.99–4.78	
Systolic blood pressure, mm Hg			<0.001			0.003
≥100	1			1		
90–99	0.85	0.61–1.18		1.05	0.58–1.98	
<90	1.57	1.17–2.13		2.36	1.32–4.19	
Primary cause of hemorrhage			0.016			0.002
Atony	1			1		
Trauma	1.40	1.05–1.88		0.93	0.52–1.66	
Placenta previa/accreta	0.91	0.63–1.32		0.47	0.22–1.02	
Other	1.54	1.06–2.23		2.37	1.31–4.29	
Nonhospital delivery	1.34	0.96–1.88	0.088	1.12	0.62–2.03	0.713
Signs of hemodynamic instability	1.76	1.25–2.47	0.001	2.60	1.24–5.43	0.008

Abbreviations: aOR, adjusted odds ratio; CI, confidence interval.

a Logistic regression adjusting for all variables in the table and random effects to correct for clustering by facility.

b Based on likelihood ratio testing.

A diagnosis of sepsis was a strong predictor for mortality in this population (*P*<0.001). Forty‐two women out of the 104 with severe sepsis died (40.4%), compared with 13 deaths out of 261 women with nonsevere sepsis (5.0%) and 428 deaths out of 19 653 women without sepsis (2.2%). In multivariate analysis, any type of sepsis was associated with a four‐fold increase in the odds of mortality (aOR 3.90; 95% CI, 2.68–5.66); however, severe sepsis increased the odds of dying 20 times (aOR 19.52; 95% 11.27–33.81) (Table 3). Mortality in women without sepsis was early (median 0.4 days after delivery, interquartile range 0.2–0.8), whereas deaths in the septic group were delayed (median 4.0, interquartile range 0.7–7.9).

**Table 3:** Effect of sepsis on mortality after adjusting for confounding.^a^

	Deaths/no. (%)	Odds ratio	95% CI	*P* value
No sepsis	428/19 653 (2.8)	1		
Any type of sepsis	55/365 (15.1)	3.90	2.68–5.66	<0.001
Nonsevere sepsis	13/261 (5.0)	0.99	0.51–1.89	
Severe sepsis	42/104 (40.4)	19.52	11.27–33.81	<0.001

a Adjusted for age, type of delivery, signs of hemodynamic instability, hospital delivery, primary cause of hemorrhage, laparotomy, intrauterine tamponade, artery ligation, hysterectomy, brace sutures, and clustering.

## 4 DISCUSSION

In this analysis of 20 060 women with PPH, invasive treatments to manage PPH such as intrauterine tamponade, hysterectomy, and laparotomy appear to increase the risk of sepsis.

Comparison with published data on sepsis is difficult because of differences in methods and definitions. However, the incidence of 1.8% in our study seems high compared with previous figures.^6^, ^17^, ^18^ This supports the hypothesis of an increased sepsis risk in women with PPH and is in line with previous studies.^8^, ^11^, ^19^ Explanations for an increased risk of sepsis include an observed cesarean delivery rate of 29.1%, which is higher than one would expect based on published national rates for cesarean delivery.^20^ Cesarean delivery is a known risk factor for both PPH and sepsis.^7^, ^12^ Moreover, hemodynamic instability is a result of PPH and causes hypoperfusion, which impairs the natural defense mechanisms of the body against infection. Nevertheless, after controlling for these and other possible confounders, the invasive management of PPH in itself appears to carry an infectious risk. In particular, hysterectomy (aOR 1.97), intrauterine tamponade (aOR 1.77), and laparotomy (aOR 6.63) appear to increase the risk of sepsis.

As consequences of sepsis are grave (15% mortality for any type of sepsis, 40% in those with severe disease) and its global impact big (11% of all maternal deaths 2 possibly contributing to more 18), prevention is of the utmost importance. We are unable to evaluate the effect of prophylactic antibiotics as their use was not recorded. Guidelines on PPH are heterogeneous in their recommendations on antibiotics.^12^, ^13^, ^14^ However, drawing on evidence for similar interventions such as surgery for spontaneous abortion**,** cesarean delivery, and hysterectomy,^7^, ^21^, ^22^ antibiotic prophylaxis seems likely to be beneficial.

Hysterectomy is known to increase the risk of sepsis, possibly due to opening the contaminated vaginal vault.^22^ The high risk associated with “laparotomy for other reasons” might be, at least partly, explained by reversed causation where second‐look laparotomies and washouts are performed for women with an intra‐abdominal infection. However, the protocol did aim to specifically record surgical interventions to treat hemorrhage. Women with PPH who undergo surgery might be more vulnerable to infection than women who have elective surgery, since they present as an emergency with possible additional risk factors (e.g. hemodynamic instability, nonhospital delivery).

Concerns about infections linked to intrauterine tamponade have been raised since the 1950s, but the currently used balloon tamponades are deemed safer in general than gauze packing. All major guidelines recommend the use of intrauterine tamponade if uterotonics are ineffective in controlling atony 12, 13, 14 and considerable enthusiasm has been generated since a 2013 literature review concluded that uterine balloon tamponade was an effective treatment in low‐resource settings.^23^ However, from the 13 reviewed studies, six were case series or case reports and none had a comparison group. Uterotonics were used concomitantly and treatment success was observed in cases with balloon volumes of only 30 mL, making it hard to assess the added benefit of tamponade. Only 241 women were included in total. A larger 2016 systematic review including 1648 women assessed the evidence for intrauterine tamponade as being insufficient owing to the small numbers of study participants and important study limitations.^24^ While there were few reported adverse events, the review notes that harms were not well characterized. However, our results raise the possibility that tamponade could increase the risk of life‐threatening sepsis. This calls for good‐quality primary research into the benefits and risks of tamponade and investigation into antibiotic prophylaxis, which is not currently part of the guidelines.

Of course, a lifesaving intervention should never be withheld to avoid the possible complication of sepsis, but clinicians should be aware of the risk and the importance of prevention, early recognition, and correct treatment.

To our knowledge, the present study is the first to look at possible explanations for an increased infectious risk in women with PPH. All recent publications that showed an association between sepsis and PPH were from studies in high‐income countries with low levels of maternal mortality 8, 9, 10, 11 or only included women who had undergone cesarean delivery.^19^ The WOMAN trial is a large multicountry study predominantly in LMICs. Recorded variables were well‐chosen for low‐resource settings and data collection was nearly complete for most of them. Severity of bleeding is likely to be an important confounder and was corrected for in our model using three different variables: estimated blood loss, systolic blood pressure, and hemodynamic instability. Plausible physiologic mechanisms, large numbers of events, and small *P* values make it highly unlikely that results are purely due to random error. Random effects were included in the model to account for clustering by facility.

There are also important limitations to consider. This was a secondary data analysis and the original study was not specifically designed to investigate the incidence or risk factors for sepsis. Clear diagnostic criteria were provided but sepsis was a secondary outcome collected from patient records. It was only measured during hospital stay. Longer admissions following surgical interventions for PPH provide more opportunity for sepsis to be diagnosed, and the effect is thus potentially overestimated. Severe sepsis was a newly created variable from sepsis and organ dysfunction, but organ dysfunction could also have been caused by hypoperfusion rather than by sepsis; therefore, misclassification of some cases is possible. In effect, the analysis of risk factors for sepsis is a cross‐sectional survey, as information on both exposure (management of PPH) and outcome (sepsis) were recorded at the same time (outcome form). Reversed causation is therefore a real danger. Sepsis can indeed cause bleeding, through a cascade of organ dysfunction leading to diffuse intravascular coagulation and abnormal clotting, and hysterectomy is sometimes performed to treat sepsis rather than causing it. Indeed, for 11% of hysterectomies the stated reason was “to remove a severely damaged, ruptured, or infected uterus.” Unfortunately, we were unable to separate out the hysterectomies performed as treatment for infection. Furthermore, “laparotomy for other reasons” might be second‐look laparotomies for abdominal infections. The original study did not collect information on comorbidities such as (gestational) diabetes or antibiotic usage, which are likely to be effect modifiers, although unable to explain the associations we saw.

In conclusion, PPH and postpartum sepsis remain important causes of maternal mortality and morbidity worldwide. In this large multicountry study, women who received certain invasive treatments to manage PPH appeared to face an increased risk of sepsis, which carried a high case‐fatality rate. Primary research is urgently required to investigate this finding further and examine ways to reduce the risk, including clearer guidelines on the use of prophylactic antibiotics. In the interim, potential harms and benefits of these interventions, particularly intrauterine tamponade, should be carefully weighed when developing policy and in clinical management of women.

## AUTHOR CONTRIBUTIONS

IR proposed the study subject. IR, HS, BF, SN, and SA were key contributors to data acquisition. IR, LC, and SW developed the study design and LC and SW analyzed the data. LC and SW drafted a first version of the manuscript, which was then critically revised by IR, HS, BF, SN, and SA. All authors provided final approval for the submitted manuscript.

## CONFLICTS OF INTEREST

The authors have no conflicts of interest to declare.

## Supporting information


**Table S1.** Guidance on diagnosing complications.
