# Supplementary material for: Secondary analysis of the WOMAN trial to explore the risk of sepsis after invasive treatments for postpartum hemorrhage
Source: Int J Gynaecol Obstet. 2019 Jun 5;146(2):231–7. doi: 10.1002/ijgo.12860 (PMC7814887; doi:10.1002/ijgo.12860)
Supplement: Supplementary file 1 — Table S1. Guidance on diagnosing complications. [file IJGO-146-231-s001.pdf]

Supplementary tables - S1 guidance on diagnosing complications

GUIDANCE FOR DATA COLLECTION

Outcome form Section 5

INTRODUCTION

This guidance is to allow standardised reporting of complications in the WOMAN trial. Before circling 'YES' to questions in Section 5 of the OUTCOME FORM, please ensure that the event fulfils the definition given below.

5. COMPLICATIONS *(circle one option on every line)*

a) Pulmonary embolism	YES	NO
b) Deep vein thrombosis	YES	NO
c) Stroke	YES	NO
d) Myocardial infarction	YES	NO
e) Renal failure	YES	NO
f) Cardiac Failure	YES	NO
g) Respiratory Failure	YES	NO
h) Hepatic Failure	YES	NO
i) Sepsis	YES	NO
j) Seizures	YES	NO

PLEASE NOTE: Any complications not listed above should be reported as per protocol using an Adverse Event Reporting form.

a) PULMONARY EMBOLISM (PE)

The diagnosis of PE requires one of the following:

- High probability ventilation/perfusion lung scan
- Intraluminal filling defect of segmental or larger artery on Helical CT scan
- Intraluminal filling defect on a pulmonary angiography

OR

- A positive diagnostic test for DVT + low or intermediate probability ventilation/perfusion lung scan or a subsegmental defect on a helical CT scan

b) DEEP VEIN THROMBOSIS (DVT)

The diagnosis of DVT requires both clinical assessment and confirmation by one of the following tests:

- A persistent intraluminal filling defect on contrast venography
- Non-compressibility of one or more venous segments on B mode compression ultrasonography
- Intraluminal filling defect on a contrast CT scan

c) STROKE

This is defined as 'a new focal neurological deficit with signs and symptoms lasting more than 24 hours'[1]

d) MYOCARDIAL INFARCTION (MI)

The universal definition of myocardial infarction[2] should be used: Detection of rise and/or fall of cardiac biomarker values (preferably troponin) with at least one value above the 99th percentile of the upper reference limit and with at least one of the following:

- Symptoms of ischaemia
- ECG abnormalities: new or presumably new significant ST-T changes or new LBBB (left bundle branch block) or pathological Q waves
- Imaging evidence of new loss of viable myocardium, or new regional wall motion abnormality
- Identification of an intracoronary thrombus by angiography or autopsy
- Cardiac death with symptoms suggestive of myocardial ischaemia
- Stent thrombosis associated with MI when detected by coronary angiography or autopsy in the setting of myocardial ischaemia and with a rise and/or fall of cardiac biomarker values with at least one value above the 99th percentile URL
